# External Load Monitoring in Female Basketball: A Systematic Review

**DOI:** 10.5114/jhk/166881

**Published:** 2023-07-15

**Authors:** Javier Espasa-Labrador, Julio Calleja-González, Alicia M. Montalvo, Azahara Fort-Vanmeerhaeghe

**Affiliations:** 1 INEFC-Barcelona Research Group on Sport Sciences (GRCE), National Institute of Physical Education of Catalonia (INEFC), University of Barcelona, Barcelona (UB), Spain.; 2 Department of Physical Education and Sport, Faculty of Education and Sport, University of the Basque Country, Vitoria, Spain.; 3 Faculty of Kinesiology, University of Zagreb, Zagreb, Croatia.; 4 College of Health Solutions, Arizona State University, Phoenix, AZ, USA.; 5 FPCEE and FCS Blanquerna, SAFE research group, Ramon Llull University, Barcelona, Spain.; 6 Segle XXI Female Basketball Team, Catalan Federation of Basketball, Esplugues de Llobregat, Spain.

**Keywords:** physical demand, dose training, team sports, woman athlete

## Abstract

The primary aim of this systematic review was to summarize the current state of research in relation to external load monitoring in female basketball. The review was conducted according to the PRISMA-P® statement. Publications included in the review: 1) were original research, 2) evaluated healthy female basketball players, and 3) monitored basketball practice and competition. The STROBE scale was used to assess quality. A total of 40 publications were included. The external load was assessed during practice (n = 9), competition (n = 11) or both events (n = 8). Also, time-motion analysis was implemented in practice (n = 2), competition (n = 9), or both events (n = 1). Accelerometry (n = 28) and time-motion (n = 12) analysis were the most frequently used methods. However, a wide range in methods and variables were used to quantify the external load. Placement of devices on the upper back and measuring with a sampling frequency of 100 Hz were most common. Player Load (PL) values increased with the competitive level of players and were higher in competition compared to training. Small-sided games can be used to gradually increase loads in female basketball (PL 5v5: 34.8 ± 8, PL 3v3: 47.6 ± 7.4, TD 5v5: 209.2 ± 35.8 m, and TD 3v3: 249.3 ± 2.8 m). Tasks without defense seemed to be less demanding. More research is needed to reach a consensus on load control in women's basketball, on what data are important to collect, and how to use and transfer knowledge to stakeholders.

## Introduction

Basketball is one of the most popular sports in the world. Its popularity has led to interest in advancing scientific knowledge of the sport, especially with the development of new technology over the last few decades (Schelling and Torres-Ronda, 2013). Baskebtball consists of aerobic and anaerobic activity during a variety of actions, such as acceleration and deceleration, change of direction, jumping or physical contact. This activity is also referred in literature as an external load (Petway et al., 2020). The external load occurs simultaneously with situations that require an understanding of the game, continuous decision-making, and anticipation of the opponents’ actions (Fox et al., 2017).

The load has two constructs: an external load and an internal load. The former refers to a dose and the latter to a response (Espasa-Labrador et al., 2021). There are three main methods used in the literature to monitor the external load: 1) video analysis, 2) positioning analysis (global and local positioning systems, GPS and LPS, respectively), and 3) accelerometry (combined with other sensors, such as magnetometer or gyroscope) (Fox et al., 2017). Most of this growing interest in the use of wearable devices to quantify the external load is focused on the latter two methods mentioned (Petway et al., 2020). Wearable devices can be used to gain an understanding of how intense an effort has been for each individual player. This allows for the evaluation of intensity and volume during a basketball game and evaluation of players’ fitness. The use of this technology has allowed sports scientists to gain a better understanding of the “dose” of activity (Petway et al., 2020). In turn, the information gained may contribute to training decisions (Vazquez-Guerrero et al., 2020), such as how to structure training plans and routines, and how to target training towards specific sport tasks. External load data can be used in combination with internal load data to understand how players adapt to training at specific points in the season (McLaren et al., 2018). While the internal load is individual and cannot be modified, the external load provides coaches with a tool to promote appropriate physiological adaptations. To achieve this goal, it is essential to determine the reference values of the external load in female basketball. By establishing reference values, coaches can accurately assess the physical demands placed on their players and tailor their training programs accordingly.

In this sense, we found systematic review publications that summarized external loading methods and physical demands values in male and female basketball players (Reina et al., 2020a; Russell et al., 2020; Stojanović et al., 2018). The above publications discussed the devices utilized, the metrics, and their limitations. Furthermore, the analyses were predominantly based on studies conducted on male players, either due to a larger sample size or an exclusive examination of only one event. Therefore, it is crucial to identify and examine the final outcome reported in publications and also consider the studied populations, which will enable us to determine the specific physical demands imposed on female basketball players during both practice and competition.

The aims of this systematic review were to summarize and compare the external load values obtained in female basketball players across different competitive age, levels, and events, during practice and competition.

## Methods

This systematic review was conducted using Preferred Reporting Items for Systematic Reviews and Meta Analyses (PRISMA-P) guidelines (Moher et al., 2016).

### Eligibility Criteria

Studies were eligible for inclusion when they met the following criteria: 1) peer-reviewed original research articles; 2) populations were healthy female basketball players of any age or level of competition; 3) research described the external load during basketball practice or competition in any format (5 versus 5 or 3 versus 3). There were no filters applied by language in order to identify all possible publications. Exclusion criteria were: 1) post-event assessment of biomechanical or neuromuscular variables; 2) validation of research instruments; 3) performance assessment; 4) wheelchair basketball studies; 5) studies that were performed for clinical purposes or therapeutic use.

### Information Sources

The search was carried out in four international databases: EBSCO, PubMed, Scopus, and Web of Science (WOS). The search was conducted through April 30, 2023.

### Search Strategy

The following search equation was used to find the relevant articles: *(“female” OR “woman”) AND “basketball” AND (“monitoring” OR “training” OR “external” OR “physical”) AND “load”*. Furthermore, the reference sections of all relevant articles were also examined, applying snowball strategy (Linder et al., 2015).

### Study Records

The search for potential publications was independently performed by two different authors (J.E.-L. and J.C.-G.). Articles were cross-referenced to identify duplicates prior to starting the screening process. An initial screening of the titles and abstracts was performed to check the eligibility criteria. When a paper could not be rejected with certainty, it was included in the eligible papers for full-text evaluation. The articles were then assessed for eligibility through a full-text screening, and those meeting the established criteria were included in the systematic review. The number of studies meeting the pre-specified inclusion criteria, and those excluded and the reasons for their exclusion were recorded and codified. All disagreements at each level were resolved by the third reviewer (A.F.-V.).

### Data Extraction

Once articles were selected for inclusion, the following data were extracted: 1) study source (author/s and year of publication); 2) type of event studied (practice or competition); 3) population of the sample, including the number or participants, mean age and the competitive level (elite, professional, amateur and youth players); 4) unit of observation (individual, team, etc.); 5) methods and devices utilized for quantification of the load (identifying manufacturer); 6) variables identified for each method; 7) outcomes reported for each variable. For time-motion analysis, additional data were extracted, including the type of motion categorized or specific actions. In those publications in which the variables to be extracted were not shown, information was requested from the corresponding author via e-mail.

The study participants were categorized into several groups: elite, professional, semi-professional, amateur and youth players. The elite group was defined by participants in the Women’s Basketball Association (WNBA), National Collegiate Athletic Association (NCAA) Division I, Euro League Women and International Basketball Association (FIBA). Professional was defined as athletes in the first and second divisions in any continent who were over 19 years old (Petway et al., 2020). The amateur level was defined as under the professional level previously mentioned. Lastly, youth competition was defined as a population that was 19 years of age or younger. Studies that analysed practice or competition through video analysis were categorized as “time-motion analysis”. Friendly games were analysed jointly with competition events while simulated games during training were included as practice events.

Final outcomes of the interventions were extracted independently by two authors (J.E.-L. and J.C.-G.) using a spreadsheet (Microsoft Excel, Microsoft Inc., Seattle, WA, USA). Subsequently, disagreements were resolved through discussion until a consensus was reached or third-party adjudication (A.F.-V.). Furthermore, the nomenclature of each original article was homogenised for better analysis of the data.

### Quality Assessment and Risk of Bias

The quality of all included studies was assessed following the Strengthening the Reporting of Observational Studies in Epidemiology (STROBE) statement: guidelines for reporting observational studies (Von Elm et al., 2007). The following scale was used to classify the study quality: 1) good quality (>14 points, low risk of major or minor bias); 2) fair quality (7–14 points, moderate risk of major bias); and 3) poor quality (<7 points, high risk of major bias). The total STROBE score was obtained through an evaluation of the 22 items of the STROBE checklist. For enhanced scientific rigour, potential study limitations were independently assessed for methodological quality and risk of bias by two authors (J.E.-L. and J.C.-G.), with any disagreements resolved by third-party evaluation (A.F.-V.).

## Results

### Study Selection

**Figure 1:**
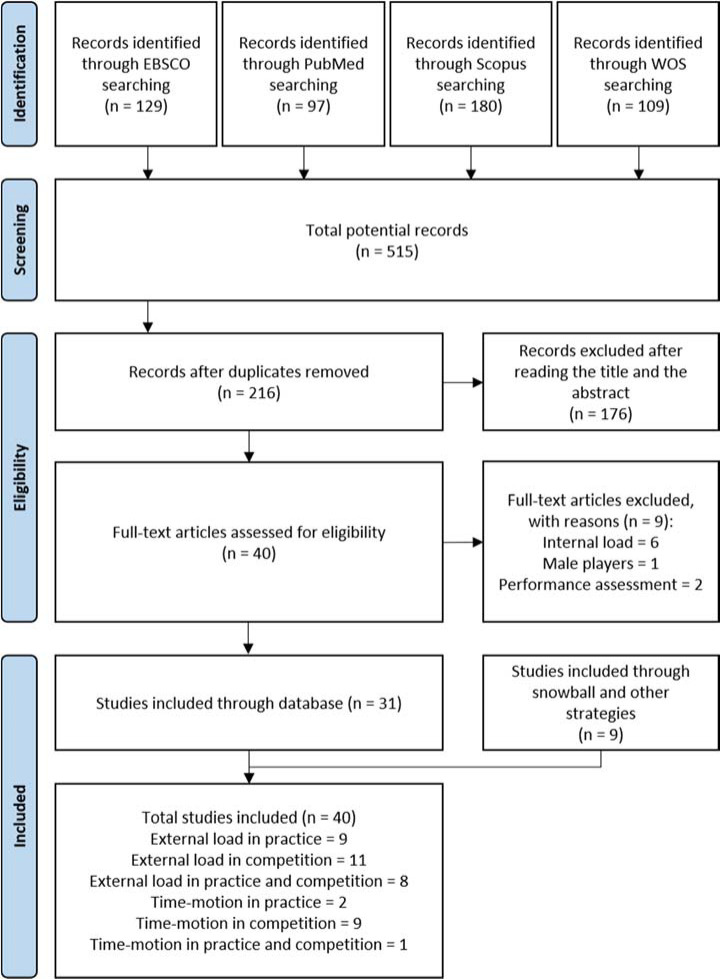
PRISMA flowchart selection of studies.

A total of 515 articles were identified in the initial search. Of the 515 articles, 299 were duplicates, resulting in a total of 216 unique articles for the title and abstract review. A total of 176 articles were removed following the title and abstract screening, leaving 40 articles to be included in the full-text assessment for eligibility. Following full-text assessment, nine articles were excluded for the following reasons: participants did not match inclusion criteria (n = 1, male players); and outcomes did not match inclusion criteria (n = 6, internal load monitoring; n = 2, performance assessment). Consequently, 31 articles met the previously defined inclusion criteria and were considered in this final systematic review. Upon completion of the snowball search strategy (Linder et al., 2015), nine more articles were included, totalling 40 articles. Figure 1 details all processes and results obtained by search strategy. A large variation was observed in variables investigated across included studies. As a result, studies were clustered by the type of the event. All results are included and summarised in tables (1–5).

### Study Participants

Samples and their characteristics are presented in Table 1. With regard to populations, youth players were monitored in 13 publications, while the remaining studies monitored adults and were categorized into adult amateur (six publications), professional (12 publications) and elite (14 publications) players (Table 1).

In the 40 included studies, there were identified two categories of load monitoring techniques: 1) wearable devices, and 2) video analysis. Of the studies included, 20 articles reported the external load during practice and 29 reported the external load during competition. Of those, nine reported external load data for both practice and competition. Studies that monitored practice sessions investigated different tasks. Tasks were categorized as follows: 1) without defense, 2) superiority task, 3) small sided game, 4) field-court session, and 5) simulated game during practice. Seven other studies investigating practice did not specify the tasks analysed. For competition, 26 studies reported external load data during a 5 *v*5 format, while three studies reported external load data during a 3 *v*3 format. Among those studies that investigated a 5 *v*5 format, only one was a friendly match, while the rest were official competition.

**Table 1:** Basic characteristics and quality assessment of studies included in this systematic review.

Publication	n	Level	Age (years)	Event	Load monitoring	Study quality (points)
Matthew and Delextrat, 2009	9	A	25.8 ± 2.5	G	IL; TM	Fair (14)
Narazaki et al., 2009	6	E	20.0 ± 1.3	P	IL; TM	Fair (14)
Delextrat and Calleja-González, 2012	9	Pro	24.3 ± 4.1	P; G	IL; TM	Fair (14)
Klusemann et al., 2012	8	Y	17.4 ± 0.7	P	IL; TM	Good (16)
Scanlan et al., 2012	12	A	22.0 ± 3.7	G	IL; TM	Good (15)
Oliveira-Da-Silva et al., 2013	96	E	27.8 ± 4.4	G	TM	Fair (13)
Conte et al., 2015	6	E	27 ± 4	G	TM	Good (17)
Delextrat et al., 2015	42	Pro	25.9 ± 4.3	G	TM	Fair (13)
Scanlan et al., 2015	12	Pro	22.0 ± 3.7	G	TM	Good (17)
Herran et al., 2017	10	Y	15.0 ± 1.0	P	EL	Fair (14)
Peterson and Quiggle, 2017	5	E	20.0 ± 1.0	P; G	EL	Good (15)
Reina et al., 2017	10	A	21.7 ± 3.7	P; G	EL; IL	Fair (13)
Staunton et al., 2018a	10	Pro	27.0 ± 5.0	G	EL	Fair (14)
Staunton et al., 2018b	9	Pro	27.0 ± 5.0	P	EL	Fair (13)
Montgomery and Maloney, 2018	208	E; Y	22.9 ± 5.6	G	EL; IL	Good (16)
Ransdell et al., 2019	6	E	19.7 ± 1.5	G	EL	Good (15)
Reina et al., 2019a	48	Y	U18	G	EL	Fair (14)
Coyne et al., 2019	12	E	27.8 ± 3.6	P	EL; IL	Good (17)
Reina et al., 2019b	10	A	21.7 ± 3.7	P; G	EL; IL	Good (18)
Reina et al., 2019c	10	A	>18	P	EL; IL	Fair (14)
Reina et al., 2019d	12	Y	U13	P; G	EL; IL	Good (15)
Portes et al., 2020	48	Y	17.0 ± 1.0	G	EL	Good (16)
Reina et al., 2020b	48	Y	U18	G	EL	Good (15)
Reina et al., 2020c	18	A	18.8 ± 2.2	G	EL; IL	Good (16)
Roell et al., 2020	12	Pro	20.7 ± 2.7	G	EL	Good (16)
Lukonaitien et al., 2020	24	E; Y	18.8 ± 0.7	P; G	EL; IL	Good (15)
Staunton et al., 2020	9	Pro	26 ± 3	P	EL; IL	Good (15)
Coyne et al., 2021	13	E	29.0 ± 3.7	P; G	EL; IL	Good (18)
Espasa-Labrador et al., 2021	13	E; Y	16.3 ± 1	P	EL; IL	Good (16)
Vencúrik et al., 2021	18	Pro	18.8 ± 1.9	G	TM; IL	Good (19)
Duque et al., 2022	32	Y	NR	P	EL; IL	Good (17)
Gutiérrez-Vargas et al., 2022	32	Y	16.2 ± 1	G	EL; IL	Good (15)
Ibáñez et al., 2022	22	Pro	22.2 ± 2.6	P	EL	Good (17)
Portes et al., 2022	48	E; Y	16.8 ± 0.7	G	EL	Good (17)
Reina et al., 2022	10	Pro	24 ± 3	P; G	EL; IL	Good (16)
Arenas-Pareja et al., 2023	14	Pro	23 ± 3.1	P	EL; IL	Good (16)
Ferioli et al., 2023a	52	E	NR	G	TM	Good (17)
Ferioli et al., 2023b	52	E	NR	G	TM	Good (17)
Towner et al., 2023	11	E; Y	20.2 ± 1.3	P; G	EL	Good (16)
Willberg et al., 2023	37	Pro	23.5 ± 4.1	G	EL; IL	Good (15)

NR: not reported; A: amateur; E: elite; EL: external load; G: game; IL: internal load; P: practice; Pro: professional; TM: time-motion analysis; Y: youth

### Outcome Variables

The clusters were external load monitoring through wearable devices during practice (Table 2) and competition (Table 3). Some studies described external load data through positioning systems such as GPS or LPS. Those publications collected different outcomes, identifying distance covered by players and how much of this distance was covered at high speed. Finally, one study showed how many sprints were detected during the event.

**Table 2:** External load method, device, and outcome during basketball practice

Publication (year) (n; level; age)	Study-defined practice mode(s)	Observations by player; Total units; Session duration (minutes)	Device (Model, manufacturer)	Outcome
Herran et al., 2017 (n = 10; Y; 15 ± 1)	FCS SSG	1–2; 5 *v*5: 10, 3 *v*3: 6; 60	10 Hz GPS (MinimaxX v.4.0, Catapult Innovations)	Outcome by variables (3 *v*3; 5 *v*5) TD: 249.6 ± 32.8; 209.2 ± 35.8, TD/min: 49.9 ± 6.6; 41.8 ± 7.2 PL: 47.6 ± 7.4; 34.8 ± 8.6, V_max_: 3.0 ± 0.4; 2.8 ± 1.1
Peterson and Quiggle, 2017 (n = 5; E; 20 ± 1)	FCS	NR; NR; NR	100 Hz tri-axial accelerometer (OptimEye S5, Catapult Sports, Melbourne, Australia)	Weekly player' average: PL: 813.1 ± 91.7, IMA: 191.0 ± 26.7 Weekly team' average: PL: 4065.4 ± 458.7, IMA: 954.9 ± 133.7
Reina et al., 2017 (n = 10; A; 21.7 ± 3.7)	SSG SG	SSG: 26, SG: 45; 122; 100	1000 Hz tri-axial accelerometer (Wimu™, RealTrack Systems, Almería, Spain)	Average outcomes (SSG; FG; 5 *v*5) Impacts/min: 2.0 ± 0.5; 1.7 ± 0.7; 1.7 ± 1.4 Steps/min: 37.5 ± 9.8; 39.2 ± 9.6; 53.5 ± 8.7 Jumps/min: 1.6 ± 0.4; 1.45 ± 0.4; 1.8 ± 0.4
Staunton et al., 2018b (n = 9; Pro; 27 ± 5)	FCS	18; 162; NR	100 Hz tri-axial accelerometer (Link, Actigraph, Pensacola, FL, USA)	AvFNet: 293 ± 40 % total duration spent in each intensity zone: Sedentary: 45.4 ± 7.7, Very light: 18.2 ± 6.1, Light: 9.8 ± 3.3 Moderate: 9.0 ± 2.4, Vigorous: 11.0 ± 3.1, Maximal: 3.0 ± 0.8, Supramaximal: 3.7 ± 1.7
Coyne et al., 2019 (n = 12; E; 27.8 ± 3.6)	NR	NR; 1717; NR	Accelerometer (NR, Catapult Sports, Melbourne, Australia)	PL/min Data shown are the product of various calculations. Direct results obtained through control methods are not reported.
Reina et al., 2019b (n = 10; A; 21.7 ± 3.7)	SG	47; 155; TT: 75.75 ± 7.74 UT: 65.42 ± 6.83	1000 Hz tri-axial accelerometer (Wimu™, RealTrack Systems, Almería, Spain)	PL/min: 0.9, Impacts/min: 1.7, Steps/min: 39.2, Jumps/min: 1.4
Reina et al., 2019c (n = 10; A; > 18)	FCS, SSG, ST, WD	120; 1200; TT: 13.8 ± 9.0 UT: 12 ± 8.4	1000 Hz tri-axial accelerometer (Wimu™, RealTrack Systems, Almería, Spain)	Average outcomes (min; max; AVG and SD) PL: 0.6; 46.8; 12.9 ± 7.9 SEL: 12.0; 1460.7; 277.4 ± 263.7
Reina et al., 2019d (n=12; Y; U13)	NR	35; 420; NR	1000 Hz tri-axial accelerometer (Wimu™, RealTrack Systems, Almería, Spain)	Steps; Jumps; PL (AU)
Lukonaitien et al., 2020 (n = 24, U20:12, U18: 12; E & Y; U20: 19.6 ± 0.8, U18: 18.0 ± 0.5)	FCS	U20: 15, U18: 18; 792; U20: 148.9 ± 59.4, U18: 146 ± 44.4	100 Hz tri-axial accelerometer (OptimEye S5, Catapult Sports, Melbourne, Australia)	Player' average outcomes (U20; U18) PL:58.9 ± 24.6; 68.0 ± 27.8, Mono-PL (AU): 4.7 ± 1.3; 5.3 ± 1.8. Strain-PL (AU): 965.5 ± 154.8; 1064.7 ± 217.7, Team' average outcomes (U20; U18) PL:706.4 ± 295.2; 816.4 ± 333.2, Mono-PL (AU): 4.7 ± 1.3; 5.3 ± 1.8, Strain-PL (AU): 965.5 ± 154.8; 1064.7 ± 217.7
Staunton et al., 2020 (n = 9; Pro; 26 ± 3)	NR	18 ; 162 ; 90-120	100 Hz tri-axial accelerometer (Link, Actigraph, Pensacola, FL, USA)	Average values by the type of a task (WU; SD; OD; DD; MS) Sedentary: 23 ± 4; 37 ± 5; 48 ± 5; 43 ± 4; 45 ± 4, Very light: 23 ± 1; 20 ± 2; 19 ± 2; 22 ± 2; 19 ± 2, Light: 16 ± 2; 11 ± 2; 9 ± 2; 10 ± 2; 9 ± 1, Moderate: 17 ± 1; 9 ± 1; 7 ± 0; 7 ± 1; 8 ± 1, Vigorous: 16 ± 1; 14 ± 1; 9 ± 1; 10 ± 1; 11 ± 1, Maximal: 3 ± 1; 4 ± 1; 3 ± 0; 3 ± 0; 3 ± 0, Supramaximal: 3 ± 1; 5 ± 2; 5 ± 2; 6 ± 1; 5 ± 2
Coyne et al., 2021 (n = 13; E; 29 ± 3.7)	NR	126.3; 1642; NR	Accelerometer (NR, Catapult Sports, Melbourne, Australia)	Total PL/min: 4.6 ± 2.0 PL/m average in practice: 4.1 ± 1.0 External weekly load: 2787 ± 772
Espasa-Labrador et al., 2021 (n = 13; E & Y; 16.3 ± 1)	FCS	35; 164; NR	Polar Pro Technology (Polar Team Pro, Polar Electro Oy, Finland)	Player' average outcomes TA: 1766 ± 10.0 TAmax: 258 ± 82.2 TA/min: 16.8 ± 2.4 Acc/min: 8.4 ± 1.2 Dec/min: 8.4 ± 1.2
Duque et al., 2022 (n = 32; Y; NR)	NR	3; NR; 90	1000 Hz tri-axial accelerometer (Wimu™, RealTrack Systems, Almería, Spain)	Player's average outcomes PL: 51.9 ± 10.0 PL/min: 1.3 ± 0.1
Ibáñez et al., 2022 (n = 22 ; Pro; 22.2 ± 2.6)	NR	10; NR; NR	1000 Hz tri-axial accelerometer (Wimu™, RealTrack Systems, Almería, Spain)	Average threshold by variable (Speed; Acc; Dec; Impacts): Very Low/Standing: < 2.3; < 0.5; > −0.4; < 1 Low/Walking: 2.3 to 5.3; 0.5 to 1.6; −0.37 to −1.1; 1 to 3 Moderate/Jogging: 5.3 to 9.3; 1.6 to 2.9; −1.1 to –2.1; 3 to 5 High/Running: 9.3 to 13.1; 2.9 to 4.3; −2.1 to –3.2; 5 to 7 Very high/Sprinting: 13.1 to 17.1; 4.3 to 6.7; −3.2 to –4.8; 7 to 10.
Reina et al., 2022 (n = 10 ; Pro; 24 ± 3)	FCS	10; NR; 120	IMU and 6 UWB antennae (Wimu™, RealTrack Systems, Almería, Spain)	Player's average outcomes TD: 2532.0 ± 962.8; TD/min: 38.5 ± 8.4; ED: 313.6 ± 118.3; ED/min: 5.1 ± 2.3; Acc: 901.3 ± 260.6; Dec: 265.0 ± 73.4; Acc/min: 15.0 ± 3.2; Dec/min: 4.7 ± 2.1; V_max_: 20.7 ± 1.6; V_avg_: 4.4 ± 0.2; Jumps: 103.4 ± 46.0; Jumps/min: 1.6 ± 0.6; PL: 41.0 ± 14.6; PL/min: 0.6 ± 0.2
Arenas-Pareja et al., 2023 (n = 14; Pro; 23 ± 3.1)	FCS	14; NR; Warm-up: 21.5 ± 8.1, Main part: 67.5 ± 14.8	8 UWB antennae (Wimu™, RealTrack Systems, Almería, Spain)	TD: 4988.0 ± 986.5; 5046.7 ± 1122.0; 6087.0 ± 469.1; 5062.1 ± 1489.4; ED: 716.4 ± 273.2; 924.5 ± 378.2; 1426.7 ± 529.5; 861.4 ± 418.1; Acc: 2514.6 ± 568.2; 2670.6 ± 865.6; 2285.4 ± 1183.9; 2454.3 ± 791,8; Dec: 2523.1 ± 569.5; 2697.1 ± 837.1; 2328 ± 1183.9; 2488.3 ± 741.0; Acc_max_: 6.8 ± 1.8; 7.8 ± 2.0; 8.3 ± 1.5; 8.1 ± 1.6; DecMax: 7.1 ± 1.8; 7.8 ± 1.8; 8.8 ± 0.9; 7.7 ± 1.6; Acc_avg_: 1.1 ± 0.2; 1.5 ± 0.6; 1.9 ± 0.8; 1.5 ± 0.7; Dec_avg_: 1.1 ± 0.2; 1.4 ± 0.5; 1.8 ± 0.8; 1.4 ± 0.6; Acc_high_: 129.4 ± 113.3; 302.9 ± 243.2; 447.6 ± 367.4; 249.5 ± 256.8; Dec_high_: 114.0 ± 90.2; 267.1 ± 216.9; 406.8 ± 322.4; 234.1 ± 229.3; TDAcc_high_: 266.3 ± 180.2; 514.3 ± 364.4; 759.2 ± 545.4; 440.5 ± 381.1; TDDec_high_: 253.7 ± 160.2; 476.5 ± 341.3; 704.3 ± 521.5; 437.3 ± 369.8; Sprints (n): 31.2 ± 18.3; 36.6 ± 20.9; 48.6 ± 22.5; 39.1 ± 21.9; V_max_: 16.4 ± 2.1; 18.6 ± 5.1; 20.6 ± 4.2; 19.0 ± 6.6; PL: 53.9 ± 10; 52.2 ± 15.6; 45.3 ± 20.1; 55.0 ± 12.4
Towner et al., 2023 (n = 11; E & Y; 20.2 ± 1.3)	NR	102; NR; NR	IMU Catapult ClearSky T6 (NR, Catapult Sports, Melbourne, Australia)	Average of the team by season’s period. These data include game data (8-h preseason; 20-h preseason; Non-Conference; Conference; Yearlong) PL: 428.7 ± 169.3; 469.9 ± 125.0; 532.2 ± 233.9; 496.4 ± 252.5; 492 ± 220.8 PL/min: 5.4 ± 2.6; 5.3 ±1.2; 5.4 ± 1.2; 5.3 ± 1.2; 5.3 ± 1.5 High-IMA: 19.7 ± 18.7; 33.8 ± 17.2; 40.3 ± 57.1; 33.8 ± 19.3; 33.7 ± 34.9 Jumps: 112.5 ± 106.7; 76.0 ± 34.7; 95.8 ± 63.3; 80.1 ± 55.3; 88.4 ± 65.6

AU: arbitrary units; NR: not reported; 3 *v*3: three versus three players; 5 *v*5: five versus five players; A: amateur; Acc: accelerations; Acc/min: accelerations per minute; Acc_avg_: average accelerations; Acc_high:_ high accelerations; Acc_max:_ maximal accelerations; AvFNet: average force net; DD: defensive drills; Dec: decelerations; Dec/min: decelerations per minute; Dec_avg_: average decelerations; Dec_high_: high decelerations; Dec_max_: maximal decelerations; E: elite; ED: explosive distance covered; ED/min: explosive distance covered per minute; FCS: full court session; FG: friendly game; High-IMA: high inertial movement assessment; IMA: inertial movement assessment; IMU: inertial movement unit; Mono-PL: monotony Player Load index; MS: match simulation drills; OD: offensive drills; PL: Player Load (AU); Pro: professional; SD: skill-development drills; SEL: subjective external load; SG: simulated game; SSG: small-side game; ST: superiority task; Strain-PL: strain Player Load index; TA: total accelerations; TA/min: total accelerations per minute; TA_max_: total maximal accelerations; TD: total distance covered; TD/min: total distance covered per minute; TDAcc_high_: total distance covered through high accelerations; TDDec_high_: Total distance covered through high decelerations; TO_high_: jump high take-offs; TT: total time; U: under age; UT: useful time; UWD: ultra-wideband system; V_avg_: average velocity; V_max_: maximal velocity; WD: without defense drills; WU: warm-up drills; Y: youth

**Table 3:** External load method, device, and outcome during basketball competition

Publication (year) (n; level; age)	Study-defined competition mode(s)	Observations by player; Total units;	Device (Model, manufacturer)	Outcome
Peterson and Quiggle, 2017 (n = 5; E; 20 ± 1)	5 *v*5 OG	NR; NR	100 Hz tri-axial accelerometer (OptimEye S5, Catapult Sports, Melbourne, Australia)	Weekly player' average: PL: 813.1 ± 91.7, IMA: 191.0 ± 26.7 Weekly team' average: PL: 4065.4 ± 458.7, IMA: 954.9 ± 133.7
Reina et al., 2017 (n = 10; A; 21.7 ± 3.7)	5 *v*5 OG	8; 80	1000 Hz tri-axial accelerometer (Wimu™, RealTrack Systems, Almería, Spain)	Impacts/min: 1.7 ± 1.4, Steps/min: 53.5 ± 8.7, Jumps/min: 1.8 ± 0.4
Montgomery and Maloney, 2018 (n = 208; E & Y; 22.9 ± 5.6)	3 *v*3 WCh 3 *v*3 ECh 3 *v*3 U18	NR 635	10 Hz GPS and 100 Hz tri-axial accelerometer (OptimEye S5, Catapult Sports, Melbourne, Australia)	TD: 856.7 ± 220.8 m; TD/min: 44.1 ± 9.6 m•min^−1^ Average by competition (WCh; ECh; U18): PL: 131.7 ± 31.2; 131.6 ± 29.7; 116.0 ± 29.0, PL/min: 6.6 ± 1.4; 6.3±1.4; 6.6 ± 1.4, VJ1: 5.3 ± 3.8; 5.0 ± 3.7; 5.3 ± 3.4, VJ2: 12.0 ± 5.3; 10.9 ± 5.2; 13.0 ± 5.8, VJ3: 2.4 ± 1.8; 2.5 ± 1.9; 2.5 ± 1.9, D1: 28.6 ± 9.1; 28.3 ± 9.8; 27.3 ± 9.9, D2: 8.8 ± 3.9; 9.1 ± 3.8; 8.8 ± 4.0, D3: 4.4 ± 2.4; 4.7 ± 2.3; 4.0 ± 2.2, A1: 20.5 ± 7.7; 21.5 ± 7.2; 18.5 ± 7.0, A2: 7.0 ± 3.2; 7.7 ± 3.4; 6.1 ± 3.3, A3: 5.6 ± 3.0; 6.2 ± 3.3; 4.3 ± 2.4, CoDL: 6.6 ± 3.2; 7.5 ± 3.8; 5.2 ± 3.4, CoDR: 4.7 ± 2.8; 4.7 ± 2.7; 4.0 ± 2.4
Staunton et al., 2018a (n = 10; Pro; 27.0 ± 5.0)	5 *v*5 OG	18; 180	100 Hz tri-axial accelerometer (Link, Actigraph, Pensacola, Florida, United State of America)	AvFNet for each zone: Sedentary: 41.9 ± 17.2, Very light: 16.7 ± 3.2, Light: 9.5 ± 4.2, Moderate: 7.6 ± 3.2, Vigorous: 11.1 ± 5.9, Maximal: 3.7 ± 1.4, Supramaximal: 5.5 ± 2.5
Ransdell et al., 2019 (n = 6; E; 19.7 ± 1.5)	5 *v*5 OG	NR; NR	100 Hz tri-axial accelerometer (OptimEye S5, Catapult Sports, Melbourne, Australia)	Average by season (PL; PL/min; High-IMA; Jumps) 2014–2015: 587.9 ± 165.3; 7.3 ± 1.0; 47.1 ± 16.5; 81.9 ± 24.0, 2015–2016: 682.7 ± 162.9; 7.4 ± 1.3; 54.2 ± 20.3; 85.8 ± 26.9 2016–2017: 678.1 ± 198.0; 6.4 ± 1.1; 52.1 ± 19.4; 92.4 ± 39.2, 2017–2018: 626.1 ± 131.1; 7.4 ± 0.9; 52.3 ± 14.2; 99.7 ± 38.2 4-year average: 655.6 ± 173.2; 7.1 ± 1.2; 52.1 ± 18.5; 89.8; 33.4
(Reina et al., 2019a) (n = 48; Y; U18)	5 *v*5 OG	3; 144	1000 Hz tri-axial accelerometer (Wimu™, RealTrack Systems, Almería, Spain)	Average by quarter (Q1; Q2; Q3; Q4) Acc: 156.3; 163.4; 158.3; 160.7 Dec: 153.0; 154.8: 148.0; 156.7 Duration of accelerations (ms): 2138.3; 2157.0; 2035.3; 2004.6) Maximal peak of acceleration (m/s^2^): 2.5, -2.6; 2.6, -2.6; 2.5, -2.6; 2.6; -2.6)
Reina et al., 2019b (n = 10; A; 21.7 ± 3.7)	5 *v*5 OG	8; 80	1000 Hz tri-axial accelerometer (Wimu™, RealTrack Systems, Almería, Spain)	PL/min: 2.8, Impacts/min: 1.7, Steps/min: 54.0, Jumps/min: 1.8
Reina et al., 2019d (n = 12; Y; U13)	5 *v*5 OG	8; 96	1000 Hz tri-axial accelerometer (Wimu™, RealTrack Systems, Almería, Spain)	NR
Lukonaitien et al., 2020 (n = 24, U20: 12, U18: 12; E & Y; U20: 19.6 ± 0.8, U18: 18.0 ± 0.5)	5 *v*5 FG	U20: 5, U18: 5; U20: 60, U18: 60	100 Hz tri-axial accelerometer (OptimEye S5, Catapult Sports, Melbourne, Australia)	Average by team (U18; U20): PL: 816.36 ± 333.19; 706.37 ± 295.2, Mono-PL (AU): 5.29 ± 1.78; 4.68 ± 1.26, Strain-PL (AU): 1064.68 ± 217.66; 965.50 ± 154.82
Portes et al., 2020 (n = 48; Y; 17 ± 1)	5 *v*5 OG	6; 288	1000 Hz tri-axial accelerometer (Wimu™, RealTrack Systems, Almería, Spain)	TD: 2513 ± 1300, HID: 237 ± 170, SprintD: 14 ± 24, Acc: 370 ± 285, Dec: 273 ± 239, Acc/min: 9.1 ± 5.3, Dec/min: 6.5 ± 3.7, Ratio Acc:Dec: 1.6 ± 0.8; PL: 39 ± 21, PL/min: 1.0 ± 0.4
Reina et al., 2020a (n = 48; Y; U18)	5 *v*5 OG	3; 144	1000 Hz tri-axial accelerometer (Wimu™, RealTrack Systems, Almería, Spain)	NR
Reina et al., 2020b (n = 18; A & Y; 3 *v*3: 17.9 ± 0.7, 5v5: 19.7 ± 3.7)	3 *v*3 OG 5 *v*5 OG	3v3: 3, 5v5: 8; 104 (3 *v*3: n = 24; 5 *v*5: n = 80)	1000 Hz tri-axial accelerometer (Wimu™, RealTrack Systems, Almería, Spain)	Average outcomes by competition (3 *v*3; 5 *v*5): Impacts: 7.5 ± 3.4; 1.7 ± 1.7, Steps: 44.4 ± 9.8; 53.3 ± 10.2, Jumps: 4.5 ± 1.9; 1.8 ± 1.0
Roell et al., 2020 (n = 12; Pro; 20.7 ± 2.7)	5 *v*5 OG	NR; NR	100 Hz tri-axial accelerometer (OptimEye S5, Catapult Sports, Melbourne, Australia)	Acc_max_ in each plane (acc_res_; acc_hor_; acc_vert_) 13.2 ± 1.3; 10.6 ± 1.3; 11.7 ± 1.2
Coyne et al., 2021 (n = 13; E; 29.0 ± 3.7)	5 *v*5 OG	12.9; 167	10 Hz GPS and 100 Hz tri-axial accelerometer, gyroscope, and magnetometer (NR, Catapult Sports, Melbourne, Australia)	Total PL/min: 4.6 ± 2.0 PL/m average in competition: 9.72 ± 1.51 External weekly load: 2787 ± 772
Gutiérrez-Vargas et al., 2022 (n = 32; Y; Pro; 22.2 ± 2.6)	5 *v*5 OG	NR; NR	8 UWB antennae and IMU (Wimu™, RealTrack Systems, Almería, Spain)	Average values according positions (guards; forwards; centers): TD (m/min): 69.2 ± 12.9; 70.8 ± 19.6; 67.4 ± 19.1, D0: 31.3 ± 4.9; 30.3 ± 6.8; 27.4 ± 6.7, D1: 26.0 ± 6.5; 26.3 ± 9.2; 23.9 ± 8.3, D2: 10.5 ± 3.7; 12.7 ± 5.4; 15.2 ± 6.4, D3: 1.2 ± 1.0; 1.3 ± 1.1; 0.9 ± 1.1, D4: 0.04 ± 0.1; 0.01 ± 0.1; 0.0 ± 0.0, Acc: 32.0 ± 13.2; 26.2 ± 3.5; 24.8 ± 3.12, Dec: 31.9 ± 13.2; 26.1 ± 3.5; 24.7 ± 3.1, V_max_: 5.1 ± 0.6; 5.0 ± 0.7; 4.8 ± 0.6, Jumps: 0.6 ± 0.3; 0.7 ± 0.3; 1.1 ± 0.6
Portes et al., 2022 (n = 48; E & Y; 16.8 ± 0.7)	5 *v*5 OG	NR; NR	100 Hz IMU and 20 Hz 6 UWB antennae (Wimu™, RealTrack Systems, Almería, Spain)	Average values according positions (guards; forwards; centers): TD: 440.7 ± 454.2; 684.5 ± 518.3; 447.3 ± 424.4, HIR: 38.9 ± 49.8; 64.7 ± 64.0; 46.9 ± 54.6, Sprint: 2.7 ± 7.8; 5.9 ± 2.0; 3.3 ± 10.0, Acc: 68.2 ± 85.0; 97.8 ± 97.1; 63.9 ± 80.0, Dec: 32.0 ± 40.7; 60.8 ± 46.6; 37.8 ± 35.2, Acc/min: 7.5 ± 7.3; 8.3 ± 6.8; 6.8 ± 6.4, Dec/min: 4.5 ± 3.7; 5.3 ± 3.5; 4.5 ± 3.8, Acc:Dec: - 0.8 ± 2.5; - 0.3 ± 2.3; - 0.15 ± 1.6, Jumps: 10.5 ± 13.3; 18.0 ± 17.9; 14.5 ± 17.2, Jumps/min: 1.2 ± 1.3; 1.5 ± 1.3; 1.6 ± 1.7, PL: 7.1 ± 7.4; 10.5 ± 8.3; 6.8 ± 6.8, PL/min: 0.8 ± 0.6; 0.9 ± 0.6; 0.8 ± 0.8
Reina et al., 2022 (n = 10 ; Pro; 24 ± 3)	5 *v*5 OG	1; NR	IMU and 6 UWB antennae (Wimu™, RealTrack Systems, Almería, Spain)	Player' average outcomes TD: 3531.6 ± 310.5; TD/min: 69.2 ± 3.0; ED: 458.7 ± 69.9; ED/min: 9.0 ± 1.0; Acc: 944.7 ± 81.4; Dec: 940.9 ± 80.1; Acc/min: 18.5 ± 1.1; Dec/min: 18.4 ± 1.1; V_max_: 19.4 ± 1.6; V_avg_: 5.2 ± 0.2; Jumps: 33.9 ± 16.0; Jumps/min: 0.7 ± 0.3; PL: 58.9 ± 9.5; PL/min: 1.2 ± 0.2
Willberg et al., 2023 (n = 37; Pro; 23.5 ± 4.1)	5 *v*5 OG 3 *v*3 OG	84; NR	100 Hz IMU (Vector Elite Vest, Catapult Sports, Melbourne, Australia) and 10 Hz UWB antennae (Vector 7, Catapult Sports, Melbourne, Australia)	Player’s average by type of event (5 *v*5; 3 *v*3): TD: 3633.1 ± 1480; 933.8 ± 219 TD/min: 70; NR, PL: 420.1 ± 162.2; 95.3 ± 25.9, PL/min: NR, EE: 35.9 ± 18.6; 14.1 ± 6.5, Jumps: 29.2 ± 15.1; 16.6 ±7.5, High-Acc/min: 8.5 ± 6.2; 3.8 ± 2.5, High-Dec/min: 5.1 ± 3.3; 3.7 ± 2.2

AU: arbitrary units; NR: not reported; 3v3: three versus three players; 5v5: five versus five players; A: amateur; A1: accelerations threshold 1; A2: accelerations threshold 2; A3: accelerations threshold 3; Acc: accelerations; Acc/min: accelerations per minute; Acc:Dec: accelerations and decelerations ratio;Acchor: horizontal maximal accelerations; Accmax: maximal accelerations; Accres: vector resultant accelerations; Accvert: vertical maximal accelerations; AvFNet: average force net; CoDL: change of direction to left; CoDR: change of direction to right; D0: distance covered between 0–6 km/h; D1: distance covered between 6–12 km/h; D2: distance covered between 12–18 km/h; D3: distance covered between 18–24 km/h; D4: distance covered at >24 km/h; Dec: decelerations; Dec/min: decelerations per minute; E: elite; ECh: European championship; ED: explosive distance covered; ED/min: explosive distance covered per minute; FG: friendly game; GPS: global positioning system; High-Acc/min: high accelerations per minute; High-Dec/min: high decelerations per minute; High-IMA: high inertial movement assessment; HIR: high-intensity-running; IMA: inertial movement assessment; IMU: inertial movement unit; Mono-PL: monotony Player Load index; OG: official game; PL: Player Load (AU); Pro: professional; Q: quarter of the game; SprintD: sprint distance; TD: total distance covered; TD/min: total distance covered per minute; U: under age; UWB: ultra-wide band indoor system; Vavg: average velocity; VJ1: vertical jump threshold 1; VJ2: vertical jump threshold 2; VJ3: vertical jump threshold 3; Vmax: maximal velocity; WCh: World championship; Y: youth

Of the articles that utilized accelerometers during practice, 14 publications used Player Load (PL) to evaluate the global physical demands, eight articles used absolute PL values, and six presented values of PL relative to time. Six publications used PL absolute and six PL relative to time values during competition. Some publications presented calculations of some indices, such as monotony and strain, through PL. Other publications studied total movement by equipping players with wrist accelerometers, showing values as the sum of movement detected, and expressing values in time spent in different intensity zones. Finally, some articles included global movement assessment inertial movement analysis (IMA) across accelerometry. Six articles were identified that provided data about total accelerations and decelerations detected during an event. Four of them presented values of the number of accelerations and decelerations related to time and one more showed calculation of a ratio between both types of actions. Moreover, other studies categorized accelerations and decelerations during different types of actions, such as jumps, steps, impacts, and changes of direction, and presented absolute values or values relative to time. Finally, some studies presented outcomes related to accelerometry, such as duration of accelerations, and peak acceleration.

Lastly, articles using time-motion analysis presented varying common actions during practice or competition including: 1) standing or walking, 2) jogging, 3) running, 4) sprinting, and 5) jumping. Some of the articles included clustered previous outcomes in three categories: 1) low-intensity shuffle, 2) medium-intensity shuffle, and 3) high-intensity shuffle. Also, three investigations described values of specific technical actions of basketball, such as dribbling or passing. All of these were summarized in Tables 4 and 5.

**Table 4:** Time-motion analysis event, sample, methods and details of analysis during basketball practice competition.

Publication (n; level; age)	Event (details)	Observations by player; Total units	Quantification external load		Details of analysis
			Device or software (manufacturer)	Variables	
Matthew and Delextrat, 2009 (n = 9; A; 25.8 ± 2.5)	Competition (5 *v*5 OG)	9; 81	Stationary immobile camera (JVC-x400, Hong Kong, China)	Jump; Sprint; Run; Jog; Stand/walk; LIS; MIS; HIS; Total of actions	Quarters analysis
Narazaki et al., 2009 (n = 6; E; 20.0 ± 1.3)	Competition (5 *v*5 OG)	6; 36	Stationary camera (ZR20, Canon U.S.A. Inc., Lake Success, New York, USA)	Stand; Walk; Run; Jump	Periods analysis; male and female comparative
Delextrat & Calleja-González, 2012 (n = 9; Pro; 24.3 ± 4.1)	Practice (SSG; DT; TT; FCS); Competition (5 *v*5 OG)	5; 45	Stationary immobile camera (JVC-x400 Hong Kong, China)	Playing game; Jump; Sprint; Run; Jog; HIS; MIS; LIS; Stand/walk; Total of actions	
Klusemann et al., 2012 (n = 8; Y; 17.4 ± 0.7)	Practice (SSG: 2 *v*2; 4 *v*4)	19; 152	Notational video analysis software (SportsCode Elite, Sydney, Australia)	Technical element: total elements, dribble, pass, close range shot, mid-range jump shot, 3-point shot, rebound ball screen External loads: Total movement, stand/walk, Jog, Run, Sprint, LIS, MIS, HIS and jump	Participants analysis; Half and full court analysis; time analysis
Scanlan et al., 2012 (n = 12; A; 22.0 ± 3.7)	Competition (5 *v*5 OG)	8; 96	Two wide-angle Baslet A602FC color cameras (Basler Vision Technologies, Ahrensburg, Germany); Labview frame-by-frame manual tracking system (National Instruments, Austin, TX, USA)	Stand/walk; Jog; Run; Sprint; LIS; HIS; Dribble; Jump*; Upper body*; Total of actions	Analysis per quarters and halves and backcourt and frontcourt
Oliveira-Da-Silva et al., 2013 (n = 96; E; 27.8 ± 4.4)	Competition (5 *v*5 OG)	4; 96	Adobe Premiere 5.0	Stand/walk; Jog; Run; LIS; MIS; Sprint; HIS; Jump; Total of actions	Position analysis
Conte et al., 2015 (n = 6; E; 27 ± 4)	Competition (5 *v*5 OG)	5; 30	Fixed camera (Sony HD AVCHD HDR-CX115, Sony, Tokyo, Japan); Dartfish software 6.0 (Dartfish, Fribourg, Switzerland)	Stand/walk; Jog; Run; Sprint; Jump; LIS; MIS; HIS	Live time, stoppage time, transfer phases, half and full court, distance of actions, with and without ball, lineal, curve and change of directions actions
Delextrat et al., 2015 (n = 42; Pro; 25.9 ± 4.3)	Competition (5 *v*5 OG)	3; 18	Video camera (25Hz); LINCE multiplatform sport analysis software (Observesport, Lleida, Spain)	Stand, Walk, Jog, Run, Sprint, Jump, LIS, MIS, HIS, Pass, Static, Total-recov, Total-low, Total-mod, Total-high, Total actions	Analysis per position and quarters
Scanlan et al., 2015 (n = 12; Pro; 22.0 ± 3.7)	Competition (5 *v*5 OG)	36; 432	Two wide-angle Baslet A602FC color cameras (Basler Vision Technologies, Ahrensburg, Germany); Labview frame-by-frame manual tracking system (National Instruments, Austin, TX, USA).	Stand/walk; Jog; Run; Sprint; LIS; HIS; Dribble; Jump*; Upper body*; Total of actions	Backcourt and frontcourt and work:rest analysis; male and female comparison
Vencúrik et al., 2021 (n = 18, U19: 10, 2^nd^ division: 8; Pro; U19: 17.6 ± 1 2^nd^ division: 20 ± 2.8)	Competition (5 *v*5 OG)	U19: 5; 50, 2nd div.: 9; 72	Video camera (streaming); Dartfish TeamPro 6.0 software (Dartfish, Fribourg, Switzerland)	Effective or ineffective dribbling and passing skills	Analysis per effectiveness, possession duration, quarters and defensive pressure
Ferioli et al., 2023a (n = 52; E; NR)	Competition (3 *v*3 OG)	NR; NR	NR; SICS VideoMatch Basket, version 5.0.5 (Bassano del Grappa, Italy)	Frequency and duration of: Stand/Walk; LIS; MIS; HIS; Sprint; High-SM; Jumps	Comparison of values between victory and defeat and between group and final phase
Ferioli et al., 2023b (n = 52; E; NR)	Competition (3 *v*3 OG)	NR; NR	NR; SICS VideoMatch Basket, version 5.0.5 (Bassano del Grappa, Italy)	Frequency and duration of: Stand/Walk; LIS; MIS; HIS; Sprint; High-SM; Jumps	Comparison of values between male and female players

NR: not reported; 2 *v*2: two versus two players; 4 *v*4: four versus four players 5 *v*5: five versus five players; A: amateur; DT: defensive task; E: elite; FCS: full court session; High-SM: high-intensity specific movements; HIS: high-intensity shuffle; LIS: low-intensity shuffle; MIS: medium-intensity shuffle; OG: official game; Pro: professional; SSG: small-side game; TT: technical task; U: under age; Y: youth

**Table 5:** Summary of physical action outcomes during competition and practice.

Competition events description in different levels								
Publication	Matthew and Delextrat, 2009 (n = 9; A; 25.8 ± 2.5)		Delextrat and Calleja-González, 2012 (n = 9; Pro; 24.3 ± 4.1)	Scanlan et al., 2012 (n = 12; A; 22.0 ± 3.7)		Oliveira-Da-Silva et al., 2013 (n = 96; E; 27.8 ± 4.4)		Conte et al., 2015 (n = 6; E; 27 ± 4)
Actions	Frequency of action; Relative frequency of action per minute		Frequency of action; Relative frequency of action per minute played	Frequency of action		Frequency of action per competition (Euroleague; WCh; Mean between both events)		Frequency of action; duration (s) of action; percentage of live time (%) spent in each action
Stand/walk	151 ± 26; 5.0		170.8 ± 61.4; 7.0 ± 1.1	436 ± 44		198 ± 9.2; 225 ± 11.22; 11.5 ± 10.2		205 ± 42; 35.4 ± 2.0; 7.42 ± 10.6
Jog	67 ± 17; 2.2		74.0 ± 14.9; 3.2 ± 0.8	551 ± 67		176 ± 15.1; 172 ± 17.9; 174 ± 16.5		73 ± 20; 12.8 ± 3.0; 2.7 ± 2.2
Run	52 ± 19; 1.7		40.3 ± 13.8; 1.7 ± 0.4	295 ± 41		231 ± 16.2; 177 ± 21.1; 204 ± 18.6		63 ± 16; 11.0 ± 1.8; 3.1 ± 1.6
Sprint	49 ± 17; 1.7		26.1 ± 15.6; 1.06 ± 0.5	108 ± 20		141 ± 21.4; 54 ± 18.0; 97.5 ± 19.7		44 ± 15; 7.8 ± 2.2; 1.8 ± 0.8
Jump	35 ± 11; 1.0		26.8 ± 18.1; 1.1 ± 0.6	43 ± 6		24 ± 3.2; 54 ± 18.0; 23 ± 6.2		19 ± 10; 3.4 ± 1.5; 0.5 ± 0.1
LIS	117 ± 14; 3.8		88.6 ± 26.8; 3.63 ± 0.4	41 ± 5		227 ± 7.8; 206 ± 14.3; 216.5 ± 11.1		91 ± 23; 15.5 ± 2.0; 1.7 ± 1.2
MIS	123 ± 45; 4.0		48.8 ± 21.1; 2.0 ± 0.5			212 ± 11; 192 ± 19.1; 202 ± 15.0		56 ± 20; 9.6 ± 2.5; 1.8 ± 1.0
HIS	58 ± 19; 1.9		28.6 ± 17.5; 1.1 ± 0.4	22 ± 5		100 ± 6.3: 50 ± 11.1; 75 ± 8.7		25 ± 10; 4.5 ± 1.5; 1.6 ± 0.9
Dribble				34 ± 2				
Pass								
Upper body				220 ± 18				
Total	652 ± 128; 21.2			1750 ± 186		1309 ± 11.2; 1098 ± 15.2; 1203.6 ± 13.2		
Publication	Delextrat et al., 2015 (n = 42; Professional; 25.9±4.3)		Scanlan et al., 2015 (n = 12; Professional; 22.0±3.7)	Vencúrik et al., 2021 (n = 18, U19: 10, 2^nd^ div.: 8; Professional; U19: 17.6 ± 1 2^nd^ div.: 20 ± 2.8)		Ferioli et al., 2023a (n = 52; E; NR)	Ferioli et al., 2023b (n = 52; E; NR)	
Actions	Relative frequency per minute; mean duration (s) per action; percentage of live time (%) spent in each action		Relative frequency per minute of action; duration (s/min); distance covered (m/min)	Frequency of action (effective; ineffective)		Frequency of action; duration of actions (% of total time) by final result and phase	Frequency of action per minute during live playing time (% total time); Frequency of action per minute during ball possession (% total time)	
Stand/walk	Stand: 6.4 ± 1.1; 2.33 ± 1.32; 30.2 ± 3.9 Walk: 2.1 ± 0.8; 2.13; 9.5 ± 4.5		10.7 ± 0.9; 21.4 ± 0.6; 11.2 ± 0.2			Win: 5.0 ± 1.6; 14.1 ± 6.4, Loss: 4.8 ± 1.7; 13.3 ± 6.5, Group: 5.0 ± 1.6; 13.9 ± 6.5, Final: 4.7 ± 1.7; 13.5 ± 6.7	5.0 (14.1); 0.2 (2.8)	
Jog	4.1 ± 1.2; 31.7 ± 5.2; 24.0 ± 9.0		13.6 ± 1.3; 21.3 ± 04; 37.4 ± 1.2					
Run	1.2 ± 0.6; 31.4 ± 4.8; 4.9 ± 2.6		7.3 ± 0.9; 10.1 ± 0.3; 45.7 ± 1.4					
Sprint	0.2 ± 0.2; 2.2 ± 0.8; 0.6 ± 0.6		2.7 ± 0.5; 2.4 ± 0.4; 22.7 ± 4.4			Win: 2.0 ± 1.0; 2.9 ± 1.4 Loss: 2.2. ± 1.1; 3.4 ± 1.8 Group: 2.0 ± 1.0; 2.9 ± 1.5 Final: 2.3 ± 1.6; 3.4 ± 1.8		
Jump	1.1 ± 0.3; 26.6 ± 3.6; 2.3 ± 1.3		1. ± 0.1; NR; NR			Win: 3.4 ± 1.0; 3.5 ± 1.1 Loss: 3.2 ± 0.9; 3.1 ± 0.9 Group: 3.4 ± 1.0; 3.35 ± 1.0 Final: 3.2 ± 0.9; 3.17 ± 0.9		
LIS	4.6 ± 2.1; 25.6 ± 3.6; 16.8 ± 8.8		1.0 ± 0.2; 1.9 ± 0.4; 1.8 ± 0.6			Win: 44.9 ± 5.9; 16.7 ± 2.8 Loss: 44.1 ± 5.8; 44.4 ± 5.8 Group: 45.4 ± 6.0; 45.4 ± 6.0 Final: 43.7 ± 5.4; 43.7 ± 5.4	15.2 (44.6); 2.0 (29.0)	
MIS	1.2 ± 1.1; 27.4 ± 4.4; 2.8 ± 2.6					Win: 16.7 ± 2.8; 16.7 ± 2.8 Loss: 17.2 ± 2.9; 17.2 ± 2.9 Group: 16.5 ± 2.9; 16.5 ± 2.9 Final: 17.4 ± 2.8; 17.4 ± 2.8	8.2 (16.8); 1.5 (20.0)	
HIS	0.3 ± 0.6; 28.1 ± 2.4; 0.7 ± 1.4		0.5 ± 0.1; 0.4 ± 0.1; 1.2 ± 0 .3			Win: 24.3 ± 4.8; 24.3 ± 4.8 Loss: 25.1 ± 4.7; 25.1 ± 4.7 Group: 24.2 ± 4.8; 24.2 ± 4.8 Final: 25.4 ± 4.6; 25.4 ± 4.6	11.9 (24.6); 4.3 (48.2)	
Dribble			0.8 ± 0.0; 2.5 ± 0.3; 8.4 ± 0.3	487; 64				
Pass				761; 153				
Upper body			5.4 ± 0.6; NR; NR					
Total	24.1 ± 3.5		44.1 ± 5.3; 2.5 ± 0.3; 128.5 ± 5.3	1248; 217		Win: 40.2 ± 3.4; NR Loss: 40.4 ± 3.4; NR Group: 40.3 ± 3.5; NR Final: 40.3 ± 3.3; NR	40.2 (NA); 8.0 (NA)	
Practice events									
Publication		Narazaki et al., 2009 (n = 6; Elite; 20.0 ± 1.3)			Klusemann et al., 2012 (n = 8; Youth; 17.4 ± 0.7)				
Actions		Frequency; Total duration by each movement			Frequency of action (4 *v*4; 2 *v*2; half-court; full-court; 2x5 min; 4x2.5 min)				
Stand/walk		Stand: 23.2 ± 13.2; 1.6±0.9 Walk: 112.0 ± 4.5; 10.6 ± 0.3			125 ± 23; 120 ± 18; 137 ± 14; 103 ± 11; 119 ± 20; 124 ± 20				
Jog					66 ± 12; 63 ± 11; 63 ± 13; 68 ± 10; 65±11; 66 ± 11				
Run		89.3 ± 0.6; 6.2 ± 0.7			35 ± 10; 35 ± 10; 34 ± 9; 37 ± 11; 33 ± 8; 38 ± 8				
Sprint					11 ± 5; 15 ± 5; 13 ± 6; 13 ± 6; 12 ± 5; 14 ± 6				
Jump		15.8 ± 5.7; 0.3 ± 0.1			16 ± 6; 26 ± 5; 23 ± 8; 18 ± 6; 20 ± 7; 22 ± 7				
LIS					42 ± 10; 39 ± 12; 45 ± 9; 32 ± 9; 39 ± 12; 40 ± 12				
MIS					75 ± 17; 72 ± 19; 81 ± 13; 62 ± 20; 69 ± 17; 77 ± 18				
HIS					8 ± 4; 13 ± 6; 11 ± 5; 7 ± 3; 9 ± 6; 12 ± 6				
Dribble									
Pass									
Upper body									
Total					378 ± 51; 382 ± 52; 407 ± 30; 340 ± 35; 20 ± 7; 22 ± 7				

NR: not reported; 2 *v*2: two versus two players; 4 *v*4: four versus four players 5 *v*5: five versus five players; A: amateur; DT: defensive task; E: elite; FCS: full court session; High-SM: high-intensity specific movements; HIS: high-intensity shuffle; LIS: low-intensity shuffle; MIS: medium-intensity shuffle; OG: official game; Pro: professional; SSG: small-side game; TT: technical task; U: under age; Y: youth

### Quality Assessment and Risk of Bias

Table 1 details the quality assessment score of each study via the STROBE tool. Studies were categorized as of good (n = 29), fair (n = 11) or poor quality (n = 0). Among the main problems in the assessment of the study quality, it was noted that: 1) ten studies adequately described the design, 2) two studies assessed bias, 3) one publication adequately detailed the sample size, and 4) two publications adequately reported changes in participants. The 11 studies labeled as of fair quality did not adequately report some aspects, i.e., a) study design, b) study limitations, c) generalization of results, and d) sources of funding. In addition, there were generally few studies that evaluated potential bias (n = 1) and sample size (n = 1).

## Discussion

The current systematic review is the first to summarize the scientific evidence available and analyse reported outcomes in relation to the external load monitoring in female basketball players. Moreover, this review includes an evaluation of the methodological quality of the included studies. The most relevant results identified in this study related to external loads will be discussed. There was great heterogeneity in methods used for monitoring external loads in female basketball players. There were also a variety of variables and values reported, which made it difficult to compare values across the included studies. During the analysis of the included studies, it was noticed that 85% of publications were from the last decade, with 70% articles published in the last five years (2018–2023). This trend coincided with a shift in research methods from time-motion analysis to the use of positioning and accelerometry devices. While this change has been previously observed in basketball research in general (Russell et al., 2020), our findings suggest that it has also occurred specifically in women's basketball. This growing interest in studying external load monitoring in women's basketball could be interpreted as an increased focus on improving athletic performance. Furthermore, this shift in methods from time-motion analysis to sensor-based technologies, such as accelerometers, may be due to their ability to provide more comprehensive and real-time data compared to video-based analyses, which can be time-consuming and resource-intensive (Russell et al., 2020). These results suggest that there may be a greater allocation of financial resources towards women's basketball research.

### External Load during Female Basketball Practice

During practice sessions, it was observed that video analysis was used only in two publications (Klusemann et al., 2012; Narazaki et al., 2009) in comparison to sensor-based methods implemented on players (such as inertial motion units, GPS, etc.). New and more sophisticated sensor-based methods allow for the quantification of both intensity and the direction of movement without requiring an evaluator to categorize particular actions (Crang et al., 2021). This not only streamlines the data collection process, but also eliminates the evaluator bias, yielding, despite its limitations (Nicolella et al., 2018), accurate values for movement magnitudes. These monitoring systems have made it possible to assess the physical demand of specific tasks as well as of the overall training session. The primary variable used to quantify external loads in most studies was Catapult’s PlayerLoad^TM^ (PL), a cumulative metric that integrates accelerations in all three axes expressed in arbitrary units (Nicolella et al., 2018).

The PL value is expressed in arbitrary units, as the sum of different movements in different directions and magnitudes. This is a “raw” value, which generates a limitation in understanding of the specific actions of basketball players, as it does not precisely identify which actions contribute most to the increase in the PL value. The data obtained from PL were treated differently by authors: 1) PL of the entire session (Lukonaitien et al., 2020), 2) PL per minute (PL/min) (Coyne et al., 2019, 2021; Reina et al., 2019a), 3) PL by task (Herran et al., 2017), 4) weekly average of PL (Peterson and Quiggle, 2017). Despite this, the authors of this systematic review want to highlight two metrics: 1) total PL of the session, and 2) PL relative to training time. The first metric helps understand the volume of training, while the second metric represents its density. Both metrics allow coaches and researchers to compare sessions globally, compare players, or even particular tasks. Regarding the latter point, interesting findings have been reported in this systematic review, as one publication showed that tasks involving defenders had a greater external load than those without defenders (Reina et al., 2019b). Regardless of this, it appears that beyond a certain level of participants, the total load decreased, which leads to a conclusion that under equal time and space conditions, 5v5 tasks were less demanding than 3 *v*3 tasks (Herran et al., 2017; Reina et al., 2019b). This could be due to the availability of space per player. A larger number of participants does not allow for covering such long distances to reach high speeds, a phenomenon already observed in male players (Bredt et al., 2020). This makes us reflect on the need to identify specific variables in 5 *v*5 situations during practice (accelerations, speed thresholds, jumps, etc.), as it has been reported in competition (Montgomery and Maloney, 2018; Portes et al., 2020). Although there is still a lack of evidence to support this approach, these initial data can help with the appropriate organization and the design of tasks during the training process, leading to optimal physiological adaptations in the preparation of the players. Moreover, the density of the workload, which expresses the relationship between work and rest, could be a better indicator of competition demands, facilitating the understanding of physiological responses (McLaren et al., 2018). In relation to the results of this review, it was observed that a higher competitive level presented a greater density of training, something that has also been observed in male players (Petway et al., 2020). However, further research is needed to establish whether density should be studied under the total time or the useful time of training (Reina, et al., 2019b).

On the other hand, some studies reported weekly average player load values, which resulted in their wide range (706.4 ± 295.2 to 816.4 ± 333.2 AU) (Lukonaitien et al., 2020; Peterson and Quiggle, 2017). Averaging several sessions over the week may not be the best way to detect acute changes in loading. Nevertheless, this measure could be useful in determining reference values for managing external loads across a season. Other publications have reported average session values per player (between 58.9 ± 24.6 and 68.0 ± 27.8 AU). This metric has similar limitations to the weekly average, although it may allow for optimizing loads in the next practice session.

### External Loads during a Basketball Game

Based on publications included in this systematic review, over half of the studies evaluated physical demands in official basketball competition. In order to accurately monitor position metrics in basketball, it is more appropriate to use the LPS than to the GPS (Russell et al., 2020). However, few studies in basketball utilized this positioning system to evaluate loads. Only one publication indicated the use of LPS in female basketball players (Portes et al., 2020). This study showed that the player’s mean total distance covered (TD) was 2513 ± 1300 m, which was similar to values in male players (Petway et al., 2020). Similarity of outcomes between different populations could be attributed to the rules of basketball that do not differ by sex, such as the lengths of possession (24 s) and the playing court dimensions (15 x 28 m). In other sports where possession of the ball is not time-dependent, there may be more differences between female and male athletes. This idea is supported by the values obtained in the 3 *v*3 competition, where values of the distance covered were much lower (856.7 ± 220.8 m) (Montgomery and Maloney, 2018). 3 *v*3 games of basketball are played on a half-court (15 x 14 m) for a maximum of 14 minutes or 21 points scored. Another metric of volume identified above was PL; however, only one publication reported such data. The study showed that averages were lower in competition compared to 5 *v*5 tasks during practice, where values were 39 ± 21 AU in youth athletes (Portes et al., 2020). These lower values and wider ranges could be explained by methodological aspects not described in the publication. Previous studies on male players recommended to only include players who played a minimum number of minutes in the data analysis (García et al., 2020). For 3 *v*3 competition, there were no differences between elite (World Championship: 131.74 ± 31.15 AU; European Championship: 131.60 ± 29.66 AU) and youth athletes (U-18: 115.95 ± 28.99 AU) with regard to the external load (Montgomery and Maloney, 2018). The similarities between competitive levels coupled with the inherent differences between 3 *v*3 and traditional basketball indicate that 3 *v*3 basketball has very specific physical demands which can be attributed to a different set of rules (space, duration and substitutes).

For intensity assessment in basketball competition, using speed thresholds-based metrics such as high-intensity-distance has been described as a valid method (Portes et al., 2020; Scanlan et al., 2015). Slightly lower values of intensity have been observed for male players compared to female ones when using the same speed thresholds (14–21 km•h^−1^) for both sexes (female players: 237 ± 170 m, and male players: 453 ± 263 m) (Portes et al., 2020). Only one another study assessed high-intensity actions, but a wide intensity range (10.8–25.2 km•h^−1^) was used, which likely resulted in the capture of very diverse actions that may have been of lower intensity (Scanlan et al., 2015). Similarly, with sprinting, there were different criteria for intensity thresholds (> 21 km•h^−1^ or > 25.2 km•h^−1^). More research is needed to establish specific intensity thresholds in female basketball players in order to better understand physical demands given the limitations discussed in this systematic review. This applies to both practice and competition. Contrary to the metrics identified in training and described previously, different actions were studied during competition: 1) accelerations and decelerations, 2) acceleration:deceleration ratio, and 3) duration of accelerations. The above-mentioned variables could be of great help in understanding the acute post-match physiological response (e.g., muscle damage/soreness) and neuromuscular status (e.g., jump power loss) in female basketball (Koyama et al., 2022). This information could guide the individualization of better training and recovery programs throughout the week. Training and recovery could be further enhanced through the analysis of additional metrics, such as steps, impacts and jumps.

During competition, it can be difficult to compare groups or to establish a reference value because of the wide variety of ways the intensity data are collected, used, and presented. Future research could focus on comparing the same group of players during different events (e.g., specific tasks or practice vs. competition) or at specific points in the season.

### Time-Motion Analysis in Female Basketball Practice and Competition

Time-motion analysis is a widespread manual notational technique to classify the movement patterns and intensities during sport events, despite its subjectivity and problems with validation and reliability (Abdelkrim et al., 2007). The ability to analyse these activities is related to the experience and reliability of the evaluator, especially those linked to movement intensity. This is an important point to consider when interpreting and applying the reference values summarized in this systematic review. Previous studies have already identified limitations with the implementation of this technique for load evaluation in basketball (Russell et al., 2020), which are primarily due to the fact that specific skills, such as dribbling or changes of direction, are not assessed in most cases.

Although this method has some limitations, it is useful for providing basic information with few resources. Semi-automated video analysis techniques exist, which simplify and optimize this process (Fox et al., 2017). The total or density of jumps per minute are examples of useful metrics, as they are easy to identify. In competition, the number of vertical actions is small, regardless of the level, with an average range of 19 to 43 jumps (Conte et al., 2015; Delextrat and Calleja-González, 2012; Matthew and Delextrat, 2009; Oliveira-Da-Silva et al., 2013; Scanlan et al., 2012), and a density close to one jump per played minute (Delextrat and Calleja-González, 2012; Matthew and Delextrat, 2009; Scanlan et al., 2015). Although all reported values could be used as a reference, it is advisable to study the specific demands in each context and adapt the training process to prepare the players for those demands. For example, it may be useful to expand the use of descriptive terms to elaborate on the type of a jumping action (e.g., bilateral, unilateral, take-off, landing, etc.). Understanding the types of jumps can be as useful in making training decisions as the overall quantity of jumps.

On the other hand, in the application of video analysis, actions where players are walking or standing can be quantified by classifying those actions as rest or standing. The observed metrics can help establish the density or a ratio between work and rest time, thus providing more information that can be used to improve the training process. In this case, the duration of these actions would be a better approximation of the density of training or competition rather than the sum of actions (Delextrat et al., 2015). Our systematic review identified that players at the highest competitive level performed more walking or standing actions during competition (from 170 to 205 occurrences) compared to athletes at lower levels. However, these findings are based on a small sample size, and more research is necessary to fully comprehend how the best basketball players manage rest moments. Possible explanations for this trend could be the higher intensity of play at the highest competitive level, which might require athletes to take short bouts of recovery between high-intensity actions (Rodríguez-Alonso et al., 2003). Additionally, athletes at higher levels of competition may have more knowledge and experience of the game, allowing them to be more efficient in their high-intensity actions and reducing the physical demand (Delextrat et al., 2015). Further investigation is necessary to confirm and expand upon the preliminary findings, which could be of significant interest as it offers valuable insights into the workload density during competition.

### Methodological Limitations and Considerations for the Future

The current systematic review presents several limitations that could largely be the consequence of varied data collection and reporting from included studies. The main limitation is related to the great heterogeneity of methods and variables used to describe volume, intensity and density variables in women's basketball, which makes the use of these data complex. We recommend evaluating the current consensus on load monitoring (Bourdon et al., 2017). Secondly, there is a lack of information on methodological aspects. The understanding of external loads, especially in practice events, would improve if more information was provided, including: 1) the number of participants in the tasks, 2) the number and duration of interruptions, 3) the size of space used, 4) active time relative to total time, etc. (Petway et al., 2020). Finally, in relation to the samples used, it is necessary to communicate how they were obtained (convenience, random, etc.), as well as their characteristics (competitive level, years of experience, etc.), and loss of participants, if necessary. Despite one of the studies aiming to measure physical demands at different stages of the menstrual cycle (Arenas-Pareja et al., 2023), none of the included publications fulfilled any of the recommended guidelines for studying female athletes. These guidelines have been established recently for a better description of samples consisting of female athletes (Elliott-Sale et al., 2021).

This systematic review showed that there was great heterogeneity in methods and variables used in external load monitoring. It is necessary to establish a consensus about methods used and data analysis to standardize volume and intensity output. Nevertheless, the literature seems to indicate that there are higher loads, through PL values, at higher competitive levels and greater frequency of high-intensity actions (e.g., jumps and sprints). At the same time, elite players spend more time standing and walking, indicating greater efficiency and intensity in actions. Total distance and high-intensity distance covered had wide ranges (TD_5_*_v_*_5_: 2513 ± 1300 m, HID_5_*_v_*_5_: 237 ± 170 m, and TD_3_*_v_*_3_: 856.7 ± 220.8 m). During practice, unopposed tasks had lower values of PL. Increasing the number of participants appeared to increase external loads. Small-sided games were the most demanding task, while 5 *v*5 practice was the least demanding (PL_5_*_v_*_5_: 34.8 ± 8, PL_3_*_v_*_3_: 47.6 ± 7.4, TD_5_*_v_*_5_: 209.2 ± 35.8 m, and TD_3_*_v_*_3_: 249.3 ± 2.8 m). Although more scientific evidence is needed to understand external loads in female basketball players, this review presents a reference for researchers and practitioners.
